# Structural Characteristics and Antioxidant Mechanism of Donkey-Hide Gelatin Peptides by Molecular Dynamics Simulation

**DOI:** 10.3390/molecules28247975

**Published:** 2023-12-06

**Authors:** Rong Liang, Le Xu, Chen Fan, Lele Cao, Xingfeng Guo

**Affiliations:** Agricultural Science and Engineering School, Liaocheng University, Liaocheng 252059, China; liangrong8209@163.com (R.L.); xule@lcu.edu.cn (L.X.); fanchen7810@126.com (C.F.); 18846170635@163.com (L.C.)

**Keywords:** gelatin peptides, antioxidant activity, structural characteristics, Keap1 protein, molecular dynamics simulations

## Abstract

This study aimed to explore the structural characteristics and antioxidant mechanism of donkey-hide gelatin peptides. After hydrolysis and ultrafiltration treatment, five gelatin peptides with different molecular weights (MWs) were obtained. Amino acid analysis showed that gelatin peptides with different MWs contained a large number of amino acids, including G, P, E, N, A, and R, and differences were noted in the content of various amino acids. Fourier transform infrared spectroscopy and circular dichroism revealed that these gelatin peptides differed in terms of the peak strength of functional groups and number of secondary structures. Moreover, 26 pentapeptides/hexapeptides were identified. Among them, we investigated by molecular docking how PGPAP, which has the best antioxidant activity, may interact with the Keap1 protein. The results showed that the PGPAP-Keap1 complex had a stable conformation, and Arg415, Gly462, Phe478, and Tyr572 were the key residues involved in the binding of the peptide PGPAP to Keap1. Our results demonstrated that PGPAP could serve as a bioactive peptide with antioxidant activity.

## 1. Introduction

Free radicals in the body can react with biologically active molecules to cause cell or tissue damage. Oxidative stress is linked to many diseases, such as inflammation and depression. Some synthetic antioxidants can scavenge free radicals and prevent food from spoiling. Although synthetic antioxidants possess strong antioxidant activity, they may cause potential harm and toxicity, which limit application in food products [1]. Therefore, the development of healthy and natural antioxidants has become a research trend. Some recent studies have demonstrated that many animal and plant protein hydrolysates have good antioxidant activities [2]. In general, an active antioxidant peptide is composed of 3–16 amino acid residues. Antioxidant peptides have the ability to scavenge free radicals, inhibit lipid peroxidation, supply hydrogen, and chelate metal ions. Considering that foodborne antioxidant peptides are safe and nontoxic, research on antioxidant peptides has gradually become a hot topic.

Gelatin is a colloidal protein extracted from the collagen of animal bones, hides, tendons, and other connective tissues. It is a straight chain polymer formed by crosslinking 18 amino acids and peptides [3]. Currently, gelatin is used as a frozen food improver, confectionery additive, beverage clarifying agent, meat product improver, dairy product additive, food coating material, and sugar coating agent in the food industry [4]. Peptides derived from gelatin possess strong antioxidant properties. For example, the EC50 values of DPPH, OH, and O^2−^ free radicals cleared by the skin gelatin hydrolysates of bluefin leatherjacket (*Navodon septentrionalis*) were 5.227, 1.147, and 4.752 mg/mL, respectively [5]. Moreover, after enzymatic hydrolysis via Protamex, these hydrolysates could scavenge DPPH free radicals and chelate Fe^2+^ ions, confirming their antioxidant effects [6].

Extensive research has proven that the antioxidant activity of peptides is affected by various physicochemical properties, including the molecular weight (MW) of peptides as well as the type and sequence of amino acids [7]. Zhang et al. revealed that the MW distribution of peanut polypeptides with the best antioxidant activity was mainly observed below 1400 Da [8]. Liu et al. obtained whey protein antioxidant peptides with different MWs and found that the peptide group with the smallest MW (<10 kDa) had the strongest antioxidant activity [9]. Sun et al. hydrolyzed porcine hemoglobin using pepsin and obtained four groups of antioxidant peptides; they revealed that that the peptide group with a MW of <3 kDa had the strongest superoxide anion scavenging ability and lipid peroxidation inhibition ability [10]. In addition, amino acid composition is a factor affecting the bioactivity of antioxidant polypeptide molecules. The enzymatic hydrolysate of smooth hound (*Mustelus mustelus*) muscle protein with strong antioxidant activity contained a high proportion of highly active amino acids, including Leu, His, Tyr, and Met [11]. Moreover, antioxidant peptides require a certain spatial structure to exert their antioxidant effects. According to a previous study, the activity of antioxidant peptides with a certain spatial structure was higher than that of amino acid mixtures with the same mass ratio [12]. However, the relationship between the bioactivity and structure of antioxidant peptides as well as the interaction between the active sites of peptide chain and amino acid residues remain unclear. Exploring the structure–activity relationship of antioxidant peptides not only helps predict the antioxidant activity of polypeptides but also provides a theoretical reference for subsequent efficient screening and synthesis of highly bioactive peptides.

Another important aspect in studying antioxidant peptides is to reveal the antioxidant mechanism. In particular, molecular docking has gradually become an important approach to screen peptides and explore their antioxidant mechanism. It is well-known that the Keap1-Nrf2 pathway is important for human cells to cope with oxidative stress. Under oxidative stress, the body can activate this pathway to promote the expression of a series of endogenous antioxidant proteins, thereby improving the body’s antioxidant capacity to resist oxidative stress [13]. Many scholars have used this pathway combined with molecular docking to explore the peptides’ antioxidant mechanism. Li et al. screened 20 small peptides with potential antioxidant activity using molecular docking, among which DKK and DDW showed the strongest ability to dock and bind to Keap1 proteins [14]. Agrawal et al. successfully isolated and purified two peptides from millet protein hydrolysates, namely, TSSSLNMAVRGGLTR and STTVGLGISMRSASVR [15]. Molecular docking studies have shown that interactions between Ser/Thr residues and free radicals are the main source of antioxidant activity of the two peptides. Tonolo et al. extracted and identified 23 peptides from fermented milk protein, among which NTVPAKSCQAQPTTM, QGPIVLNPWDQVKR, and APSFSDIPNPIGSENSE showed a strong ability to dock and bind to Keap1 [16]. These three peptides have certain inhibitory effects on Keap1-Nrf2 PPI; moreover, they can upregulate the expression level and activity of antioxidant enzymes by activating the Keap1-Nrf2 pathway [17]. These studies provide a significant reference for exploring the antioxidant mechanism of active peptides.

This study aimed to explore the structure–activity relationship of donkey-hide gelatin peptides using amino acid analysis, Fourier transform infrared (FTIR) spectroscopy, and circular dichroism (CD). In addition, gelatin peptides were identified, and the antioxidant peptides with a known sequence were synthesized. The antioxidant activity of gelatin peptides was analyzed, and the most active peptides were docked to the Keap1 protein through molecular docking. Moreover, the stability of the peptide–Keap1 complex was verified using molecular dynamic (MD) simulations to explore its antioxidant mechanism. This research may indicate the molecular mechanism of active peptides and provide a reference for application of gelatin peptides.

## 2. Results and Discussion

### 2.1. Antioxidant Activity of Gelatin Peptides with Different MWs

**Figure 1:**
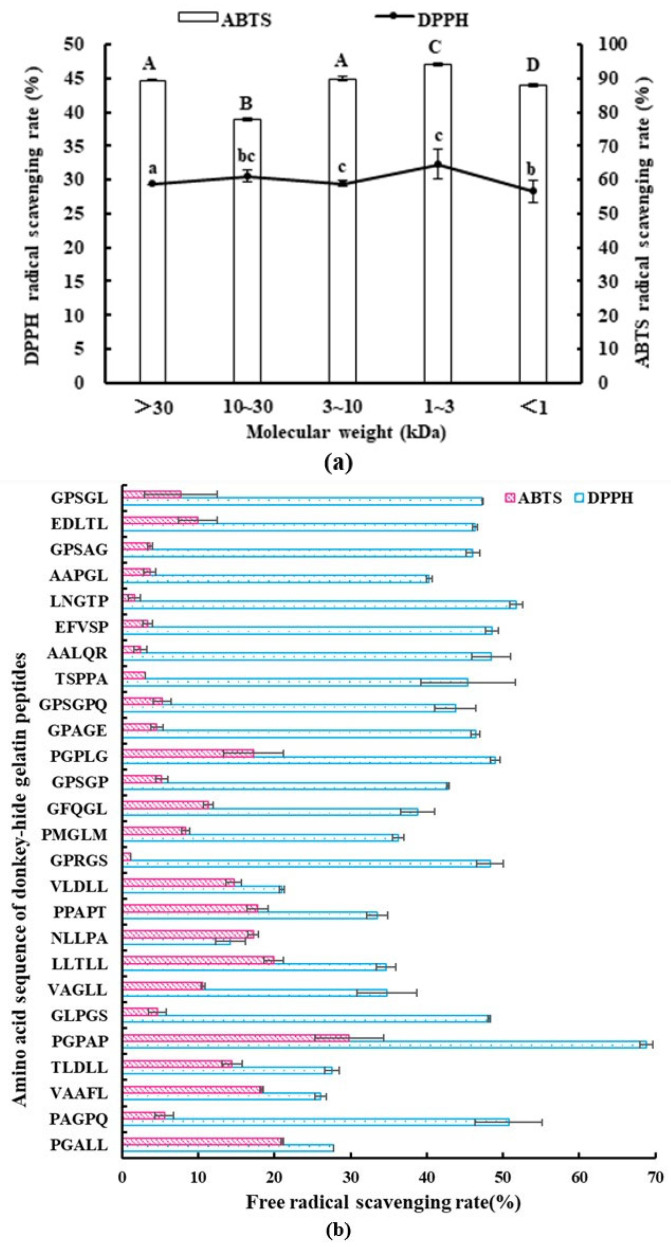
Antioxidant activity of donkey-hide gelatin peptides. (**a**) DPPH and ABTS scavenging ability of donkey-hide gelatin peptides with different molecular weights. The same capital letters A–D mean that the variance of ABTS scavenging ability between two samples is not significant (*p* > 0.05), and the different letters A–D mean significant (*p* < 0.05); the same small letters a–d mean that the variance of DPPH scavenging ability between two samples is not significant (*p* > 0.05), and the different letters a–d mean significant (*p* < 0.05). (**b**) DPPH and ABTS scavenging ability of donkey-hide gelatin peptides with different amino acid sequences.

After enzymatic hydrolysis and ultrafiltration of donkey-hide gelatin, five MW groups of gelatin polypeptides were obtained, including those with a MW of >30, 10–30, 3–10, 1–3, and <1 kDa. Then, the antioxidant activity of gelatin polypeptides was tested. The results are shown in Figure 1a. Peptides with an MW of 1–3 kDa had the highest antioxidant activity, with DPPH and ABTS free radical scavenging abilities of 32.33% ± 2.19% and 94.13% ± 0.37%, respectively. A previous study reported that the antioxidant activity of peptides with a certain spatial structure was higher than that of amino acid mixtures with the same mass ratio [12], and the smaller the MW of peptides, the higher the antioxidant activity. However, in this study, peptides with the smallest MW (<1 kDa) did not show the highest antioxidant activity, which may be due to the presence of more free amino acids in their system. Ngoh et al. also obtained consistent results by separating and purifying pinto protein-hydrolyzed products; in other words, the peptide group with a MW of <3 kDa had the strongest antioxidant activity [18].

### 2.2. Amino Acid Composition of Gelatin Peptides with Different MWs

The antioxidant activity of peptides is closely related to their structural properties, such as MW, amino acid composition, and peptide configuration. To explore the structure–activity relationship, the amino acid composition, functional groups, and secondary structure of polypeptides with different MWs were detected and analyzed. The amino acid composition (a total of 17 amino acids) of each polypeptide group was analyzed (Table 1). Many amino acids, such as G, P, E, N, A, and R, were identified in each group of peptide hydrolysates. In addition, each group contained a certain amount of M, C, and H. The amino acid composition of a peptide has a crucial effect on its antioxidant activity, and some amino acids may be detected in the active sites of antioxidant peptides [19]. A previous study revealed that the E-L sequence contributed significantly to the free radical scavenging ability of the short antioxidant peptide YFYPEL [20,21]. Chi et al. (2015) reported that YLMSR and VLYEE had higher antioxidant activity than MILMR, which might be related to the presence of Y [22]. Saito et al. revealed that peptides containing Y had stronger antioxidant activity than those containing H; moreover, peptides containing W or Y at the carboxyl terminal showed stronger free radical scavenging ability [23]. In this study, although the amino acid types were identical among the peptide groups, differences were noted in the contents of various amino acids. For example, the contents of most of the amino acids (e.g., N and E) in the group with a MW of >30 kDa were higher because the overall amino acid content was higher in this group of large proteins, which are not completely hydrolyzed. However, the contents of G and I were the highest in peptides with a MW of 1–3 kDa. The difference in amino acid composition among different groups may be responsible for their different antioxidant activities.

### 2.3. Functional Groups and Secondary Structures of Gelatin Peptides with Different MWs

**Figure 2:**
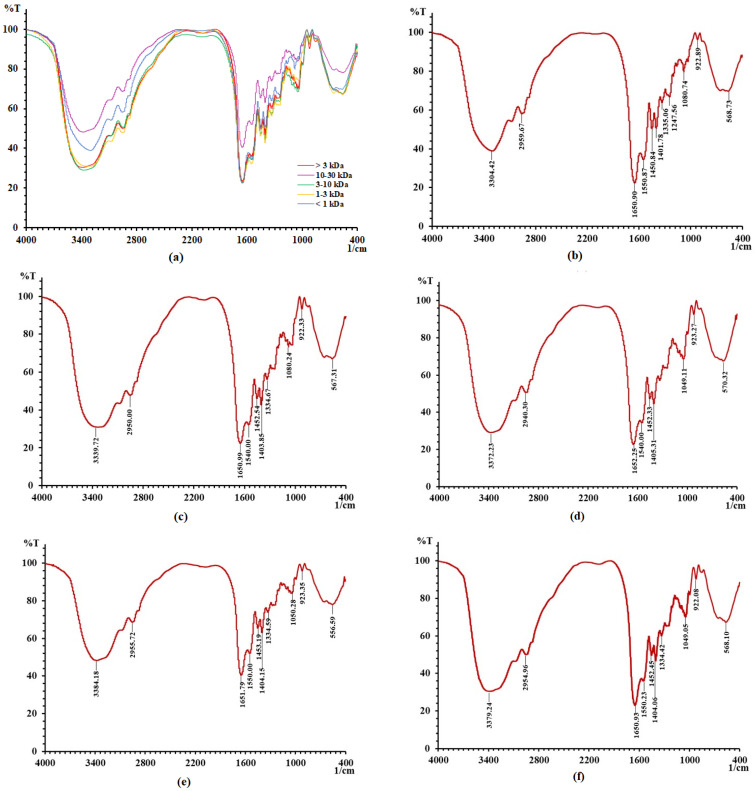
The FTIR spectra of donkey−hide gelatin peptides with different molecular weights. (**a**) FTIR spectra of all donkey−hide gelatin peptides, (**b**) FTIR spectra of donkey-hide gelatin peptide with MW of <1 kDa, (**c**) FTIR spectra of donkey-hide gelatin peptide with MW of 1–3 kDa, (**d**) FTIR spectra of donkey-hide gelatin peptide with MW of 3–10 kDa, (**e**) FTIR spectra of donkey-hide gelatin peptide with MW of 10–30 kDa, and (**f**) FTIR spectra of donkey−hide gelatin peptide with MW of >3 kDa.

As shown in Figure 2, all gelatin peptides with different MWs showed absorbance in N–H (3500–3300 cm^−1^), O–H (3300–2500 cm^−1^), unsaturated C–H (>3000 cm^−1^), saturated C–H (3000–2800 cm^−1^), –C=O (1850–1600 cm^−1^), amide (1700–1600 cm^−1^), C=C (1680–1620 cm^−1^), C–N (1360–1180 cm^−1^), C–O (1300–1080 cm^−1^), S=O (1220–1040 cm^−1^), C–O–C (1150–900 cm^−1^), and –NO_2_ (1600–1500 cm^−1^) bands. Some of these bonds were found in amino acids, while some do not exist in amino acids such as S=O and –NO_2_, which may produce absorption peaks due to stretching between amino acids. Gelatin peptides possessed the same absorption bands because they came from the same source and had the same amino acid composition. However, the peak strength of functional groups varied in peptide groups with different MWs (Figure 2a). This finding may be related to the different amino acid contents, indicating that the spatial structure of the polypeptide aqueous solution is different. It is certain that antioxidant peptides require a spatial structure to exert their antioxidant effects. Differences in spatial structure can affect the exposure of certain active groups and thus affect the antioxidant activity of polypeptides [12].

**Figure 3:**
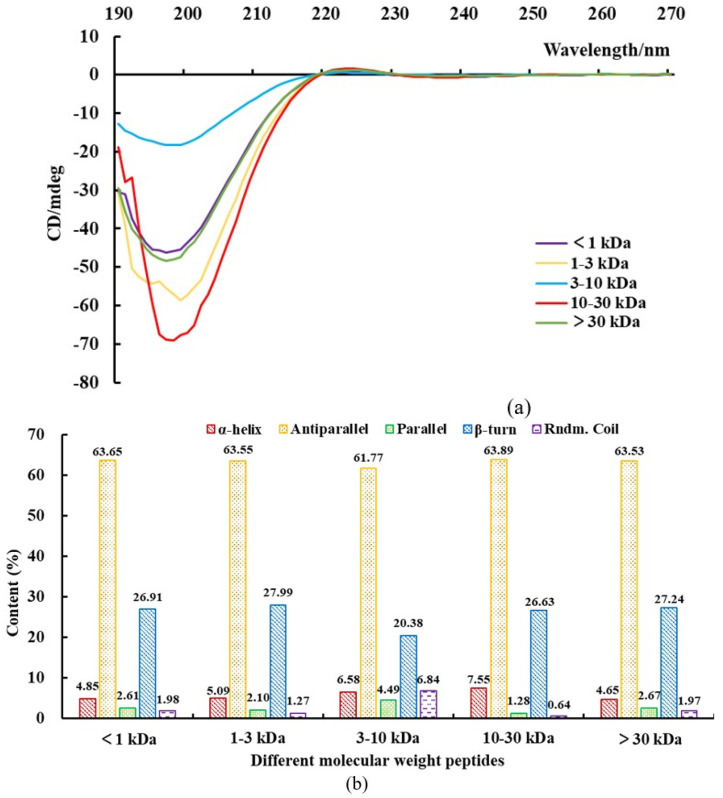
Secondary structure results of donkey−hide gelatin peptides with different molecular weights. (**a**) Circular dichroism spectrum, and (**b**) secondary structure content.

All CD spectra of gelatin peptides with different MWs showed a negative peak at approximately 200 nm, which is considered a p–p band (Figure 3a). In addition, a small positive band was observed at approximately 225 nm. Based on the CD spectra, poly-proline II helix and random coil conformations were detected, with different ellipticity values at wavelengths below 220 nm indicating different content of conformations [24]. As shown in Figure 3b, gelatin peptides presented a high content of antiparallel conformations, followed by beta-turn, whereas random coil, parallel, and alpha-helix conformations were relatively rare. Although gelatin peptides with different MWs showed similar trends in the content of different secondary structures, some differences were also noted. These subtle differences can affect the antioxidant activity. For example, a small change in random coil conformation can influence the antioxidant activity [25]. Antioxidant peptides require a certain spatial structure to exert their antioxidant effects. The best free radical scavenging ability can be achieved when some special groups associated with antioxidant activity are fully exposed [26]. There may be some oligopeptides in gelatin peptides solution, which could not form secondary conformations alone, but some oligopeptides with a very short length may exist in the form of peptide complexes to exhibit a secondary structure [27]. Therefore, differences in the secondary structure of gelatin peptides is another factor affecting antioxidant activities.

### 2.4. Sequence Identification and Antioxidant Activity of Synthetic Peptides

To identify the key sequence responsible for antioxidant activity in gelatin polypeptides, LC–MS/MS analysis of the polypeptide group with a MW of 1–3 kDa was performed, and 26 pentapeptides/hexapeptides were identified (Table 1b). These peptides were derived from seven different proteins, including eight peptides from obscurin, cytoskeletal almodulin, and titin-interacting RhoGEF; seven from collagen alpha-2 type I chain; five from ubiquitin protein ligase E3 component n-recognin 4; two from the SZT2 subunit of the KICSTOR complex; two from neurobeachin-like 2; one from dynein heavy chain domain 1; and one from zinc finger homeobox 4. In addition, the higher the overall score of peptide identification, the more reliable the identification results. The identified peptides were synthesized chemically, and their antioxidant activities were analyzed via DPPH and ABTS scavenging ability assays (Figure 1b). The top five peptides showing DPPH scavenging ability included PGPAP, LNGTP, PAGPQ, PGPLG, and EFVSP, whereas those showing ABTS scavenging ability included PGPAP, PGALL, LLTLL, VAAFL, and PPAPT. PGPAP showed the best antioxidant activity, with DPPH and ABTS scavenging abilities of 68.83% ± 1.84% and 29.82% ± 4.51%, respectively. Previous research reported that the antioxidant activity of Leu-Leu-Pro-His-His (soy protein peptide) decreased after the removal of C-terminal His, whereas no effect was observed on antioxidant activity when the N-terminal Leu was removed [28]. Moreover, His and Pro play an important role in the antioxidant activity sequence because the peptide Pro-His-His has high antioxidant activity, and Tyr does not enhance the activity when introduced at the Pro or His location. In addition, the N-terminal of the maize antioxidant peptide contains the hydrophobic amino acid Leu, which enhances the interaction between antioxidant peptide and fatty acid and improves its ability to trap lipid free radicals [29]. In this study, most peptides with higher antioxidant activities contained Pro-like PGPAP and PAGPQ. Some peptides contained the hydrophobic amino acid Leu at the N-terminal, such as LNGTP and LLTLL, with high ABTS scavenging ability.

### 2.5. Molecular Dynamics (MD) Simulations and Stability Analysis of PGPAP Binding to Keap1

**Figure 4:**
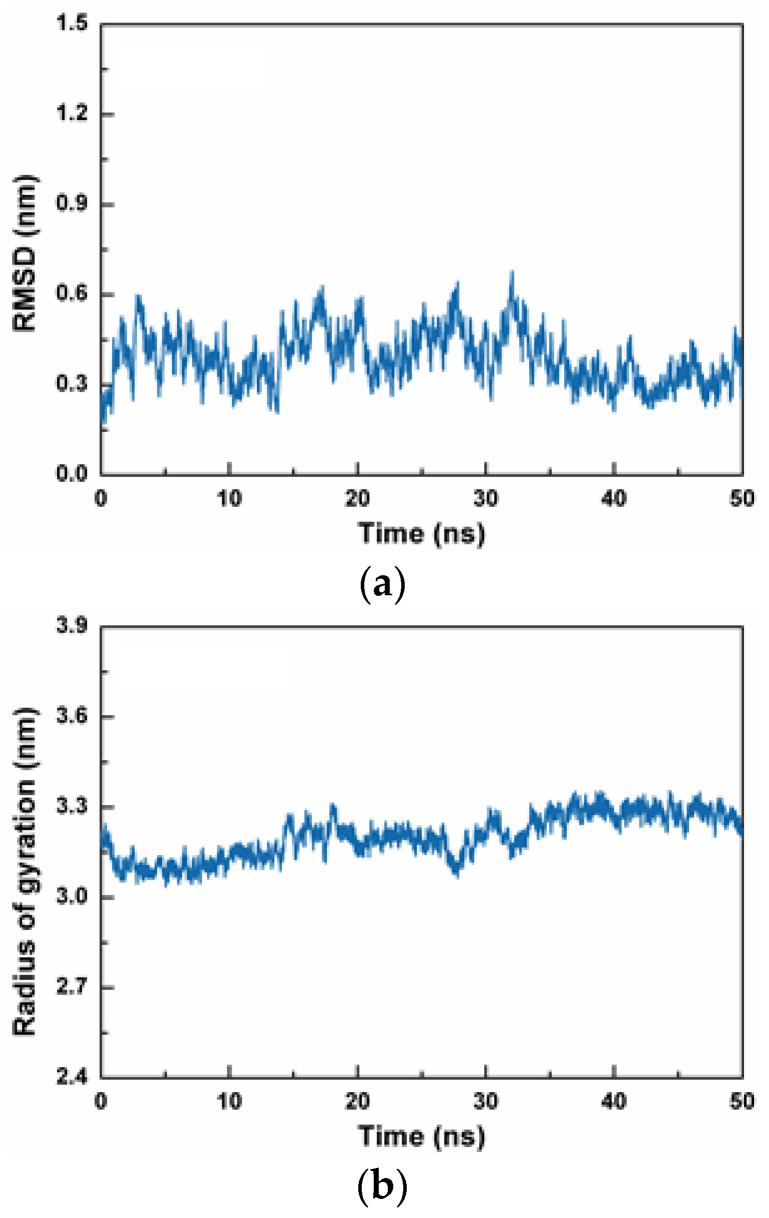

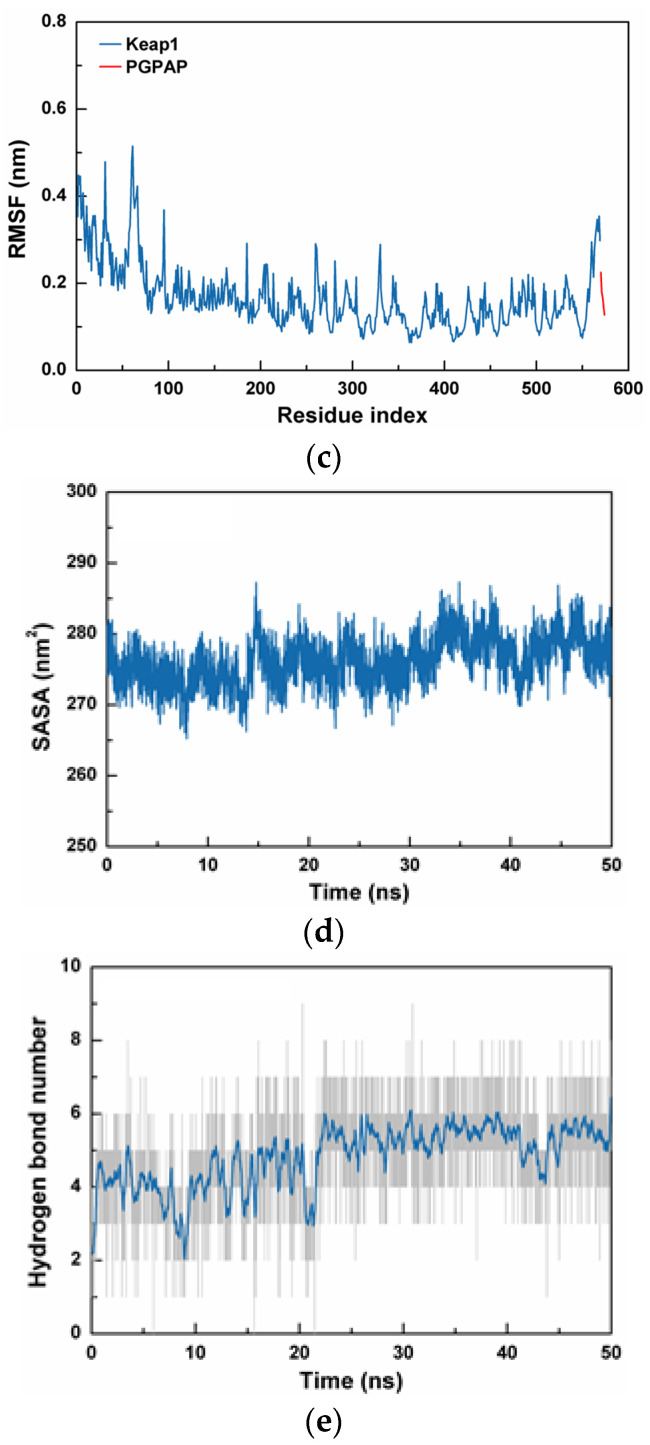
Root-mean-square deviation (RMSD), radius of gyration (Rg), root-mean-square fluctuation (RMSF), solvent-accessibility surface area (SASA) and hydrogen bond number curves of the protein backbone (Cα) atoms. (**a**) RMSD curves of the PGPAP-Keap1 complex. (**b**) Rg curves of the PGPAP-Keap1 complex. (**c**) RMSF curves of the PGPAP-Keap1 complex. (**d**) SASA curves of the PGPAP-Keap1 complex. (**e**) Hydrogen bond number curves of the PGPAP-Keap1 complex.

The PGPAP, which showed the best antioxidant activity, was docked onto Keap1 protein, to explore the antioxidant mechanism of gelatin peptides. Using AutoDock, the predicted binding energy of PGPAP to the Keap1 protein was determined to be −5.375 kcal/mol. Then, stability of PGPAP binding to Keap1 was characterized by MD simulations of 50 ns length. Root-mean-square deviation (RMSD) is an important parameter for measuring the stability of the system. Figure 4a shows the changes in the RMSD values of all atoms in the PGPAP–Keap1 complex system over time. The RMSD of the complex increased significantly at the initial stage of simulation. Subsequently, the RMSD of the system began to stabilize at approximately 20 ns, and the average RMSD of the whole process was 0.389 ± 0.086 nm. Therefore, the dynamic simulation based on RMSD was stable and reliable, which could be used for further analysis.

Radius of gyration (Rg) indicates the compactness of the protein’s overall structure [30]. If protein folding is stable, then Rg can maintain a relatively stable value, and a large change in Rg indicates that the system is more expansive. As shown in Figure 4b, the Rg of the whole system decreased at the beginning of the simulation and then gradually increased. The whole complex interacts with solvent molecules, leading to a certain expansion of the protein structure, followed by complete stabilization at approximately 30 ns. The Rg value of the PGPAP–Keap1 complex during simulation reached approximately 3.202 ± 0.072 nm. This finding indicated that the structure of the PGPAP–Keap1 complex was compact and stable.

Root-mean-square fluctuation (RMSF) indicates the flexibility of amino acid residues in a protein. As shown in Figure 4c, the RMSF value of PGPAP indicated that the whole protein structure was stable during simulation and the polypeptide molecule did not alter greatly after binding to proteins. Thus, it was determined that such a molecule could stably bind to the active pocket of the Keap1 protein.

The solvent-accessibility surface area (SASA) was used to characterize the molecular surface area of the protein system, which may be in contact with the solvent. Consequently, the structural volume changes of proteins in a solvent were analyzed. A high SASA value indicates a loose protein structure and large volume. The SASA value of the PGPAP–Keap1 complex did not increase or decrease significantly during simulation, and the mean value of SASA was 276.219 ± 3.312 nm^2^ during the whole process (Figure 4d). The small fluctuation indicated that the complex formed by the peptide and Keap1 protein remained stable throughout the dynamic simulation. Therefore, PGPAP could tightly bind to Keap1 in stable conformations, thereby confirming its potential application as an antioxidant peptide.

### 2.6. Molecular Mechanism Analysis of Peptide PGPAP Binding to Keap1

Variations in the number of hydrogen bonds between the Keap1 protein and PGPAP polypeptide were analyzed (Figure 4e). The number of hydrogen bonds between them decreased slightly when the whole PGPAP–Keap1 complex underwent conformational change and stabilized, and the number of hydrogen bonds increased further. The average number of hydrogen bonds between Keap1 and PGPAP was 5.36 when the RMSD value was stable at 25–50 ns. Consequently, PGPAP polypeptide molecules could stably bind to Keap1 protein.

**Figure 5:**
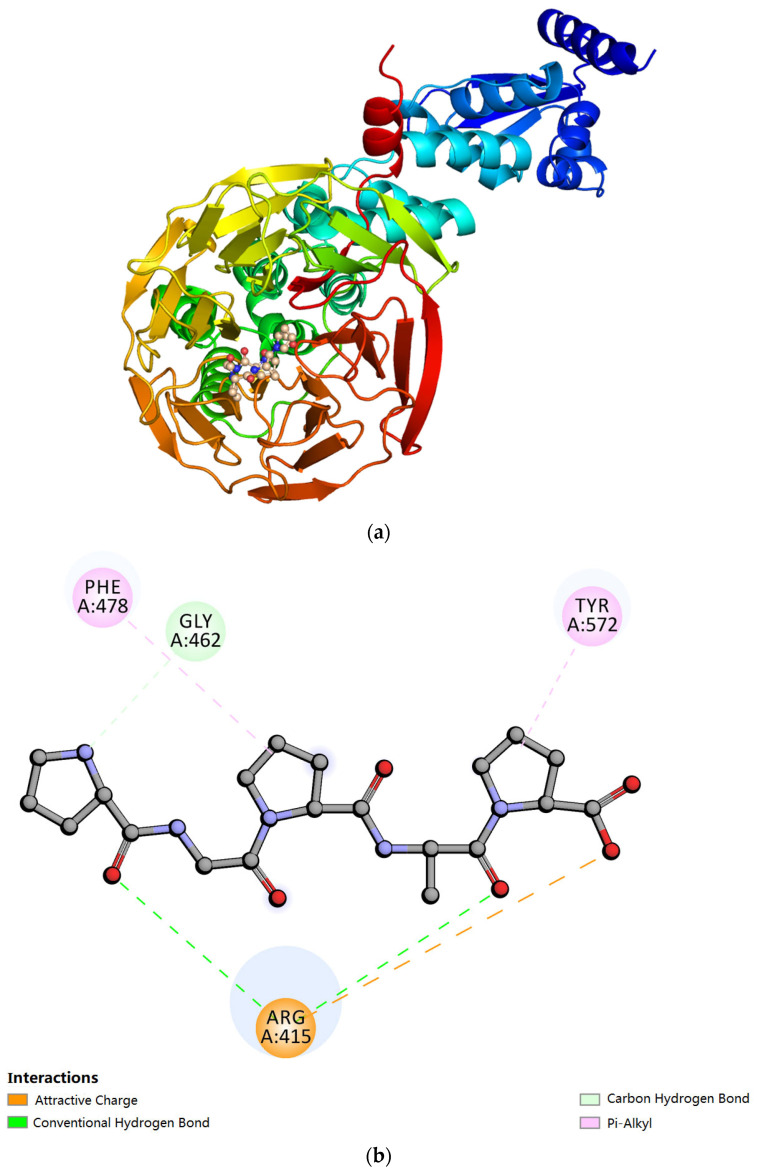

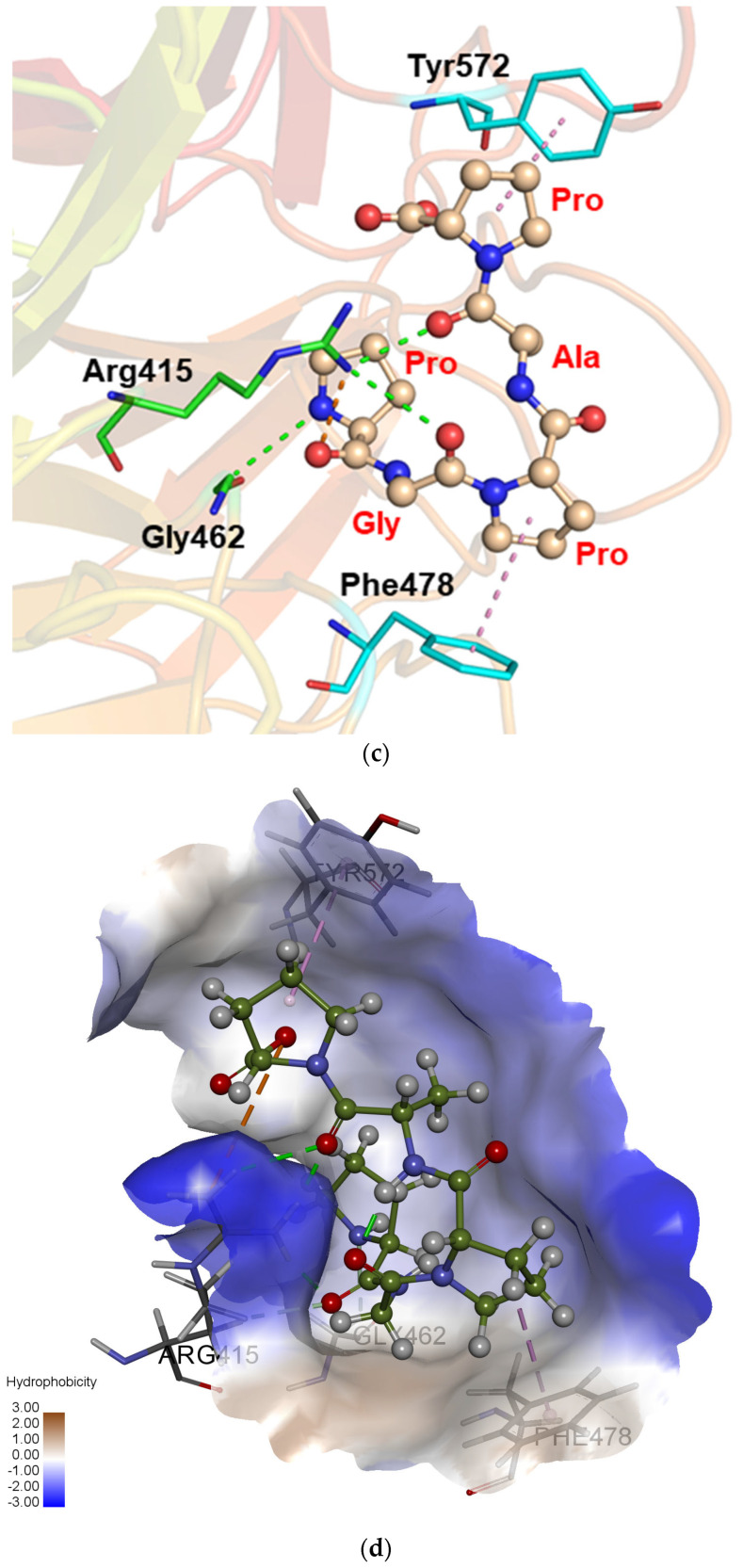
Docking interactions of PGPAP with Keap1. (**a**) Crystal structure of Keap1 bound with PGPAP, (**b**) 2D diagram how PGPAP interacts with Keap1. The dotted green lines represent hydrogen bonding, orange represent salt bridge interactions, and pink represent π–π stacking interaction. (**c**) 3D diagram how PGPAP interacts with Keap1, and (**d**) hydrophobic interfaces of PGPAP binding to Keap1. Blue and gray colors represent the hydrophilic and hydrophobic parts of the protein surface, respectively.

This study extracted the complex conformation of polypeptides after MD simulation and analyzed the interaction among them to confirm the main interaction regions between Keap1 and PGPAP as well as key amino acids involved in the interaction. As shown in Figure 5a, the polypeptide molecules are primarily bound to the hydrophobic cavities of the Keap1 protein. Based on the analysis shown in Figure 5b,c, the O atom (carboxyl oxygen from amide bond of the peptide PGPAP), which acts as the hydrogen bond acceptor formed a hydrogen bond with the surrounding amino acids like Arg415 and Gly462. Meanwhile, the polypeptide molecule also formed a π–π stacking interaction with the benzene ring on the surrounding amino acids—Phe478 and Tyr572—which further enhanced the affinity of the polypeptide molecule towards the Keap1 protein. The results indicated that the peptide PGPAP can stably bind to the active cavity of the Keap1 protein to play the corresponding biological role. In addition, the hydrophobic interfaces between PGPAP and Keap1 are shown in Figure 5d. The peptide PGPAP was bent and folded into the hydrophobic pocket of Keap1. The residues Gly462 and Phe478 were responsible for forming the PGPAP-induced hydrophobic interface, which helped the polypeptide molecules enter the active pocket and stably bind to the Keap1 protein.

The human Keap1 protein has five domains, namely, N-terminal region, POZ region (also known as the BTB region), IVR region, KELCH region, and C-terminal region [31]. The human Nrf2 protein can be divided into six conserved domains, namely, Neh1, Neh2, Neh3, Neh4, Neh5, and Neh6 [32]. The Neh2 region is responsible for binding to the Keap1 protein. Under normal physiological conditions, Nrf2 is continuously produced in cells. Keap1 binds to cytoplasmic actin through its POZ region to form homologous dimers; moreover, it binds to the DLG and ETGE sequences of the Neh2 region of Nrf2 protein through two Klech regions of the dimer [33]. In addition, the Keap1 protein dimer binds to Cul3 through its POZ region, which may promote the ubiquitination of the Nrf2 bound to the Keap1 protein dimer, thereby leading to the degradation of ubiquitinated Nrf2 and ensuring that free Nrf2 proteins in the body are present at low levels [34]. When the cells are under oxidative stress, foreign oxidants can alter the conformation of key cysteine residues in POZ and IVR regions of Keap1, thereby altering the conformation of the Keap1 dimer. This conformational change may interfere with the Keap1–Nrf2 interaction. If foreign antioxidants affect the Keap1–Nrf2 interaction, which may increase the intracellular Nrf2 content, then such substances can activate the pathway and improve the antioxidant capacity of the body. In this study, MD simulation suggested that PGPAP could stably bind to Keap1, indicating that it has good potential antioxidant function. Further experiments are warranted to explore its intracellular antioxidant activity and mechanism using HepG2 cell models.

## 3. Materials and Methods

### 3.1. Materials and Reagents

Donkey-hide gelatin (batch number: 2110014) was purchased from Dong-E-E-Jiao Co., Ltd. (Liaocheng, China). Alcalase 2.4L was obtained from Novozymes Biotech. Co., Ltd. (CPH, Denmark). Potassium bromide (KBr), potassium persulfate (K_2_S_2_O_8_), formic acid, acetonitrile, 2, 2-diphenyl-1-picrylhydrazyl (DPPH), 2, 2-azino-bis (3-ethylbenzothiazoline-6-sulfonic acid) diammonium salt (ABTS), and amino acid standard (AAS18) were obtained from Sigma Chemicals Co. (USA). All other chemical reagents were of analytical grade purity and obtained from Peking Chemical Plant (Beijing, China).

### 3.2. Preparation of Gelatin Peptides with Different MWs

Gelatin peptides were prepared according to the method of Yu et al. [35]. Briefly, 5 g of donkey-hide gelatin was dissolved in 120 mL of water and then simmered in a water bath at 80 °C for 30 min. After cooling at 63 °C, the pH of the donkey-hide gelatin solution was adjusted to 10.5 using NaOH (1 mol/L), and Alcalase 2.4L (enzyme to substrate ratio of 9%) was added to initiate hydrolysis. During enzymolysis, the pH range was maintained within ±0.05. After hydrolysis for 3 h, the enzyme was inactivated in a water bath at 90 °C for 10 min, followed by centrifugation of gelatin hydrolysates for 10 min at 10^4^ r/min and 4 °C. Then, the supernatant was filtered through Amicon ultra centrifugal filters (Millipore, Burlington, MA, USA) with interceptions of 30, 10, 3, and 1 kDa. Finally, the gelatin-derived peptide solutions with different MWs were obtained.

### 3.3. Analysis of the Amino Acid Composition of Gelatin Peptides

Overall, 100 mg of gelatin peptide powder was placed in a hydrolytic tube, and 10 mL of HCl (6 M) was added to the tube in a ratio of 1:1. Nitrogen was blown into the tube for 30 s, after which the tube was sealed. Then, the sample was hydrolyzed in an oil bath at 110 °C for 24 h. After hydrolysis, it was cooled to room temperature and filtered through a 0.45-μm membrane into a 50-mL volumetric bottle. Next, the samples were deacidified and filtered through a 0.45-μm filter for machine analysis using Biochrom 30+ amino acid analyzer (Biochrom Ltd., FCE, Cambridge, UK). An Na-type cationic resin chromatography column (200 mm × 4.6 mm) was used for gradient elution. The temperature of the separation column was 55 °C–65 °C–77 °C, and the temperature of the reaction tank was 138 °C. The flow rate of the buffer solution was 20 mL/h, whereas that of the reaction solution was 10 mL/h. The sample volume was 50 μL, and the ultraviolet (UV) detection wavelengths were 570 nm and 440 nm.

### 3.4. Analysis of the Functional Groups of Gelatin Peptides via FTIR Spectroscopy

FTIR spectroscopy was performed with a resolution of 4 cm^−1^ over the range of 4000–400 cm^−1^ using a UV spectrometer (Thermo Nicolet iS5, Thermo Fisher, Waltham, MA, USA). Briefly, 2 mg of peptide powder and 200 mg of dried KBr were mixed to prepare the sample pellets. The pellets were placed in a sample compartment and measured 32 times at a scanning speed of 2.8 mm/s. After background correction (dried KBr spectrum used as background), the sample spectra were obtained.

### 3.5. Analysis of the Secondary Structure of Gelatin Peptides via CD

The CD spectra and secondary structures were analyzed using Chirascan 100 Circular Dichroism Spectropolarimeter (Applied Photophysics Ltd., FCE, Surrey, UK). Before the detection test, the nitrogen flow rate was adjusted to between 0.15 and 0.2 MPa; nitrogen was passed for 30 min, and the gas in the optical path of the instrument was removed. The gelatin peptide powder was mixed with deionized water to form a liquid sample (1 mg/mL), which was then slowly poured into the sample pool with an optical path of 1.0 cm for the test. The wavelength range was set from 190 to 270 nm; optimal bandwidth was set to 1 nm; scanning speed was set to 100 nm/min; and the scan time was set to three accumulations. Finally, the data were analyzed using the following equation [36]:

$$[\theta ]=\frac{\theta }{10LCN}$$

where *θ* indicates instrument readings, *L* indicates the path length (cm), *C* indicates concentration of the peptide (mol/L), and *N* indicates the number of amino acid residues in the peptide.

### 3.6. Determination of the Antioxidant Activity of Gelatin Peptides

#### 3.6.1. DPPH Radical Scavenging Assay of Gelatin Peptides

The reaction system comprised of 96-well microplates containing 100 μL of gelatin peptide solution (6 mg/mL), 100 μL of fresh DPPH (0.6 mM), and 100 μL of methanol. Subsequently, samples were shaken vigorously and incubated at 25 °C for 30 min under dark conditions. Then, the absorbance was measured at a wavelength of 515 nm (using methanol as a blank). Finally, the DPPH radical scavenging activity of gelatin peptides was estimated using the following equation:

$$DPPH\;radical\;scavening\;activity\;(\%)=(1-\frac{Asample}{Ablank})\times 100$$

#### 3.6.2. ABTS Radical Scavenging Assay of Gelatin Peptides

The ABTS radical scavenging assay was performed in accordance with the method of Kozics et al. with minor modifications [37]. Overall, 10 mL of ABTS (7 mM) and 10 mL of K_2_S_2_O_8_ (2.45 mM) were mixed under dark conditions for 16 h to generate ABTS radical cations. Then, 50 μL of gelatin peptide solution (6 mg/mL) or deionized water (as blank) was added to 150 μL of diluted ABTS solution (absorbance of 0.70 ± 0.02 at 734 nm) in a 96-well microplate. Subsequently, the solution was left to incubate for 6 min under dark conditions, and the absorbance was measured at a wavelength of 734 nm. The ABTS scavenging activity of gelatin peptides with different MWs was calculated as follows:

$$ABTS\;radical\;scavening\;activity\;(\%)=(1-\frac{Asample}{Ablank})\times 100$$

### 3.7. Peptide Sequence Identification Using Liquid Chromatography (LC)–Tandem Mass Spectrometry (MS/MS)

A Zorbax 300SB-C18 peptide trap (Agilent Technologies, Wilmington, DE, USA) was used to separate the gelatin peptides, and while a RP-C18 capillary column was used as the analytical column (0.15 mm × 150 mm; Column Technology Inc., Mississippi, USA). Separation was performed using a mobile phase consisting of 0.1% (*v*/*v*) formic acid aqueous solution (A) and 0.1% (*v*/*v*) formic acid acetonitrile solution (B), according to the method of Yu et al. [38]. The elution conditions were set as follows: 0–50 min, 4% B → 50% B; 50–54 min, 50% B → 100% B; and 54–60 min, 100% B. After the separation of gelatin peptides via capillary high-performance LC, they were analyzed via MS using a Q Exactive mass spectrometer (Thermo Fisher, Mass, USA). The analysis duration was set to 60 min, and detection was performed in the positive-ion mode. Ten fragment maps (MS2 scan) were collected after each full scan. Finally, the raw mass spectrum file was uploaded to MaxQuant 1.5.5.1 to obtain peptide sequences based on the database UniProt_Equus_asinus_33662_20221212. The peptide tolerance was set to 20 ppm, MS/MS tolerance was set to 0.1 Da, and false discovery rate of peptides and proteins was set to ≤0.01.

### 3.8. Peptide Synthesis

The antioxidant peptides with known sequences were synthesized by Hubei Qiangyao Biotech Co., Ltd. (Hubei, China), using Fmoc-protected amino acid synthesis.

### 3.9. Antioxidant Activity Tests of Synthetic Peptides

The antioxidant activity of synthetic peptides was tested using DPPH and ABTS radical scavenging assays, as described in Section 2.6. The concentration of synthetic peptides was determined to be 2 and 1 mg/mL according to DPPH and ABTS assays, respectively.

### 3.10. Molecular Docking of Gelatin Peptides with Keap1

The Keap1 protein with a three-dimensional crystal structure (obtained by ab initio modeling with AlphaFold2 [39]) was used as the initial receptor for molecular docking, and the PGPAP polypeptide was a straight chain structure directly constructed via Pymol [40]. The random coil part (1–55 amino acid residues) with very low modeling quality was deleted in the Keap1 sequence. This structure section is far from the overall active structure of the protein and has no impact on the biological function of the whole protein. The polypeptide and Keap1 were connected globally, and the sites with the strongest binding effect were selected for analysis. The binding site of the polypeptide molecule in a protein is primarily located in the cavity above the center of the barrel structure, which is composed of a large number of β-sheets at the C-terminus of the protein. Therefore, the active docking site of the polypeptide and protein was selected in this pocket. Molecular docking was implemented using AutoDock 4.2.6, and the center coordinates of the docking box were set as follows: x = 14.219, y = 1.850, and z = −21.613. The number of cells in each XYZ direction was set to 60 × 60 × 60, and the frequency of docking was set to 100 times. The docking was carried out by Lamarckian genetic algorithm in Autodock. The whole docking process adopted a semi-flexible method; that is, the receptor was regarded as rigid and the ligand as flexible. An independent docking run was performed during the molecular docking of gelatin peptides with Keap1. In this docking run, the protein and the small molecule were docked 50 times. An Amber14 force field was adopted for energy optimization [30]. It was carried out in two steps: firstly, the structure was optimized by the steepest descent method with 1000 steps, and then further optimized by the conjugate gradient method with 500 steps.

### 3.11. Molecular Dynamic (MD) Simulations of the PGPAP-Keap1 Complex

Gromacs 2018.4 [30] was used to run MD simulations of the PGPAP-Keap1 complex using the Amber14SB force field [41] and TIP3P water model [30]. During MD simulation, all bonds involving hydrogen atoms were constrained using the LINCS algorithm, and the integral step was 2 fsc. The electrostatic interaction was calculated using the particle-mesh Ewald method. The nonbond interaction cutoff value was set to 10 Å and was updated every 10 steps. The simulated temperature was controlled at 298.15 K using the V-rescale temperature coupling method, and the pressure was controlled at 1 bar using the Parrinello–Rahman method.

### 3.12. Statistical Analysis

One-way analysis of variance was used to perform triplicate analyses using SPSS 13.0 software (SPSS Inc., Chicago, IL, USA). All data were presented as the mean ± standard. A *p*-value of < 0.05 was considered to indicate statistical significance.

## 4. Conclusions

This study investigated the structural characteristics and antioxidant mechanism of donkey-hide gelatin peptides. The DPPH and ABTS free radical scavenging activity assays indicated that gelatin peptides with a MW of 1–3 kDa had the strongest antioxidant activity. Amino acid analysis, FTIR spectroscopy, and CD revealed that gelatin peptides with different MWs differed in terms of amino acid composition, peak strength of functional groups, and content of secondary structures, which might result in different antioxidant activities. In addition, molecular docking and MD simulation indicated that PGPAP could bind to the Keap1 protein at the following key residues: Arg415, Gly462, Phe478, and Tyr572. The PGPAP–Keap1 complex exhibited stable and compact conformations, which appear a plausible basis to exert its biological function. Overall, the antioxidant activity of gelatin peptides is closely related to their structural properties, and PGPAP may serve as an antioxidant peptide targeting in vivo metabolism.

## Figures and Tables

**Table 1:** (a). Amino acid composition of donkey-hide gelatin peptides with different molecular weights. (b). Amino acid sequence identification of donkey-hide gelatin peptides (MW < 1 kDa).

**(a)**						
**No.**	**Amino Acid Type**	**Amino Acid Content of Donkey-Hide Gelatin Peptides (mg/g)**				
		**>30 kDa**	**10–30 kDa**	**3–10 kDa**	**1–3 kDa**	**<1 kDa**
1	Asn (N)	73.189	63.137	61.07	60.278	50.186
2	Thr (T)	29.513	25.766	27.857	26.285	23.272
3	Ser (S)	39.896	34.747	34.871	35.864	33.833
4	Glu (E)	107.354	90.651	86.697	88.686	78.885
5	Gly (G)	128.919	118.312	124.697	129.384	115.45
6	Ala (A)	63.607	60.372	56.137	59.293	59.261
7	Cys (C)	11.416	11.579	15.318	14.41	9.948
8	Val (V)	25.303	23.089	21.879	22.12	21.671
9	Met (M)	23.675	22.583	20.419	21.044	20.839
10	Ile (I)	32.001	31.48	33.731	35.121	29.606
11	Leu (L)	36.751	33.759	32.873	35.278	32.613
12	Tyr (Y)	16.381	13.858	14.759	15.526	12.439
13	Phe (F)	28.169	26.427	26.334	25.897	24.547
14	His (H)	10.438	9.922	9.249	9.64	8.856
15	Lys (K)	43.891	41.852	39.377	39.551	39.326
16	Arg (R)	60.535	55.137	44.898	51.678	50.074
17	Pro (P)	113.935	96.871	92.763	95.652	96.794
**(b)**						
**No.**	**Amino Acid** **Sequence**	**Leading Razor Protein**	**Protein Names**		**Gene Names**	**Score**
1	GPSGL	A0A8C4MG85	Obscurin, cytoskeletal calmodulin and titin-interacting RhoGEF		OBSCN	61.28
2	EDLTL					57.71
3	GPSAG					52.86
4	AAPGL					25.12
5	LNGTP					25.08
6	EFVSP					24.47
7	AALQR					24.30
8	TSPPA					20.54
9	GPSGPQ	B9VR89	Collagen alpha-2 type I chain		COL1A2	162.47
10	GPAGE					63.06
11	PGPLG					55.36
12	GPSGP					50.40
13	GFQGL					32.63
14	PMGLM					26.57
15	GPRGS					22.12
16	VLDLL	A0A8C4MF50	Ubiquitin protein ligase E3 component n-recognin 4		UBR4	43.99
17	PPAPT					38.19
18	NLLPA					33.54
19	LLTLL					23.49
20	VAGLL					22.99
21	GLPGS	A0A8C4L3F1	SZT2 subunit of KICSTOR complex		SZT2	56.75
22	PGPAP					61.37
23	TLDLL	A0A8C4LPB4	Neurobeachin like 2		NBEAL2	45.11
24	VAAFL					28.74
25	PAGPQ	A0A8C4KVK0	Dynein heavy chain domain 1		DNHD1	81.36
26	PGALL	A0A8C4MZE5	Zinc finger homeobox 4		ZFHX4	25.12

## Data Availability

Data are contained within the article.

## Abbreviations

Molecular weights, MW; 2, 2-Diphenyl-1-picrylhydrazyl, DPPH; 2, 2-azino-bis (3-ethylbenzothiazoline-6-sulfonic acid) diammonium salt, ABTS; Fourier transform infrared, FTIR; Circular dichroism, CD; Liquid chromatography−tandem mass spectrometry, LC−MS/MS; Molecular dynamic, MD; Potassium bromide, KBr.
